# Intravoxel Incoherent Motion Diffusion-Weighted MR Imaging can Differentiate between Residual or Recurrent Hepatocellular Carcinoma and Inflammatory Reaction Zone: A Single-Center Study

**DOI:** 10.2174/0115734056468200260622070740

**Published:** 2026-07-07

**Authors:** Yuchang Yan, Xuechao Du, Yufei Lian, Erhu Jin, Zhenyu Pan

**Affiliations:** 1 Department of Radiology, Beijing Chaoyang Hospital, Capital Medical University, Beijing 100043, China; 2 Department of Radiology, Beijing Friendship Hospital, Capital Medical University, Beijing 100050, China

**Keywords:** Diffusion magnetic resonance imaging, Hepatocellular carcinoma, Radiofrequency ablation, Recurrent HCC, Post-RFA tumors, Inflammatory reaction zone

## Abstract

**Introduction:**

The aim of this study was to explore the application of IVIM-DWI (intravoxel incoherent motion diffusion-weighted imaging) MRI (magnetic resonance imaging) in the evaluation of abnormal enhancement of the margin of hepatocellular carcinoma (HCC) after radiofrequency ablation (RFA).

**Materials and Methods:**

We performed a routine IVIM-DWI imaging review of patients who had been diagnosed with HCC and had received RFA in the past four years. We made quantitative comparisons of D, D*, and f in different tissues and lesions, and used one-way analysis of variance to compare quantitative data and an ROC (receiver operating characteristic) curve to analyze the diagnostic efficacy of the data.

**Results:**

A total of 79 patients met the study’s criteria. The consistency of the measured values of the three surveyors was very good (ICC > 0.90). The D of recurrent HCC and the inflammatory reaction zone was (0.65 ± 0.16) × 10^−3^ mm^2^/s and (0.87 ± 0.05) × 10^−3^ mm^2^/s, respectively. The D* of recurrent HCC and the inflammatory reaction zone was (31.34 ± 11.54) × 10^−3^ mm^2^/s and (41.37 ± 5.11) × 10^−3^ mm^2^/s, respectively. The f of recurrent HCC and the inflammatory reaction zone was (18.16 ± 5.82)% and (24.21 ± 3.69)%, respectively. The differences in IVIM-DWI parameters between the two groups were all statistically significant (*P* < 0.001).

**Discussion:**

Our research indicated that the D of recurrent HCC was lower than that of the inflammatory reaction zone. The optimal cutoff value can differentiate between these two types of lesions. Our research revealed a statistically significant difference in D* between recurrent HCC and the inflammatory reaction zone. The ROC curve shows that this optimal cutoff value has high diagnostic efficiency. The f is higher in hepatic parenchyma and inflammatory reaction zones than in other tissues. Therefore, f may help distinguish the inflammatory reaction zone from recurrent HCC.

**Conclusion:**

IVIM-DWI is valuable for differentiating between regions of recurrent HCC and the inflammatory reaction zone in post-RFA tumors. In terms of diagnostic performance, D outperformed D* and f.

## 1 INTRODUCTION

According to the European Association for the Study of Liver Diseases, liver cancer ranks second [1] in cancer-related deaths worldwide. Hepatocellular carcinoma (HCC) accounts for approximately 75% to 85% of primary liver cancer [1, 2]. Only about 15–20% of cases of HCC are amenable to surgical resection [3]. Radiofrequency ablation (RFA) is currently the most commonly used treatment for liver cancer in the world [4]. European and American guidelines recommend RFA as a primary treatment for patients with early-stage HCC who cannot undergo liver surgery or transplantation [5, 6]. Research shows that the recurrence of liver cancer post-RFA is between 14.1% and 58.1%. Detecting recurrent tumors early and performing RFA can enhance the complete ablation rate to 77%–99.7% [7]. Consequently, it is vital to accurately assess the efficacy of RFA to improve the prognosis for those with HCC [8].

The inflammatory reaction zone around the HCC RFA lesion is heat-mediated inflammatory edema or granulation hyperplasia. The inflammatory reaction zone, which can be significantly enhanced during the arterial phase of the enhanced scan, is similar to the recurrent tumor signal in the non-enhanced MR sequence. For this reason, it is sometimes difficult to distinguish the inflammatory reaction zone from recurrent HCC. In previous studies, the edge of the ablation margin was assessed by comparing axial CT-MRI images before and after radiofrequency ablation using a side-by-side approach. These approaches are subjective, and their ability to accurately assess the ablation margin is limited [9, 10]. In addition, it is often necessary to follow up to confirm the differentiation between tumor recurrence and the inflammatory reaction zone, which may lead to missing the best treatment opportunity.

IVIM-DWI can simultaneously quantify changes in water molecule diffusion and microcirculation perfusion, thereby quantitatively evaluating the functional status of HCC [11]. IVIM-DWI has been widely used in the study of liver diseases [12, 13]. Studies have shown that IVIM-DWI can differentiate between solid and cystic lesions in the liver, as well as between malignant and benign liver tumors [12]. An animal experiment demonstrated that the D and f values were significantly lower in viable tumor regions than in inflammatory reaction regions [14]. As far as we are aware, there is no research employing IVIM-DWI to differentiate the inflammatory reaction area from recurrent tumor after RFA in human HCC.

The objective of our investigation is to determine whether IVIM-DWI can accurately distinguish abnormal enhancement at the HCC margin after RFA and provide an early and accurate evaluation of RFA effectiveness.

## 2 MATERIALS AND METHODS

### 2.1 Patient Population

**Fig. (1):**
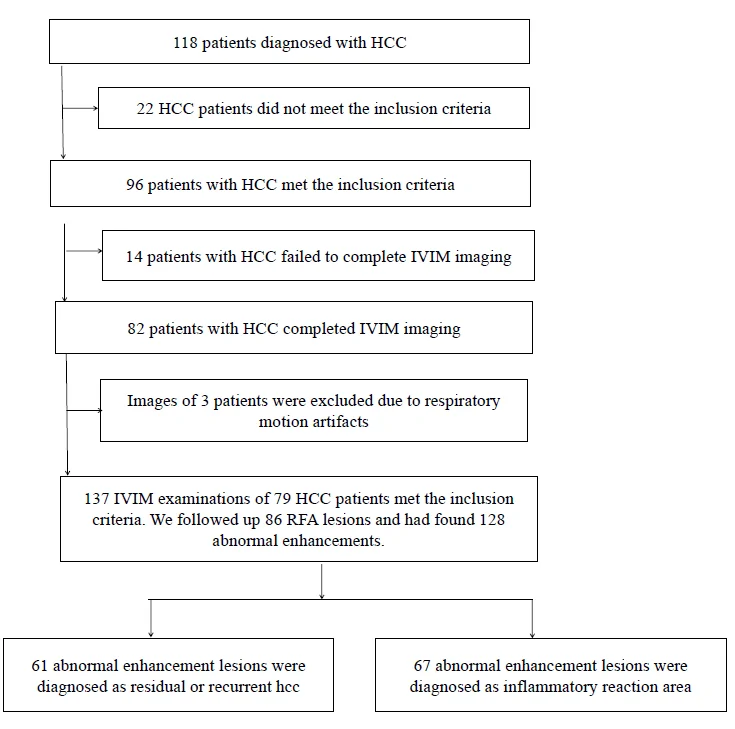
The flowchart shows the study selection process based on inclusion and exclusion criteria. **Abbreviations**: HCC=hepatocellular carcinoma, IVIM=intravoxel incoherent motion, RFA=radiofrequency ablation.

Our study was performed in accordance with the principles of the Declaration of Helsinki and its appendices. The study was approved by the Ethics Committee of Beijing Friendship Hospital, Capital Medical University (ID: 2015-Scientific Research-28), with written informed consent obtained from all patients. This study is prospective. Patients with HCC treated at the author’s unit between December 2015 and March 2019 were included (Fig. **1**). Regular magnetic resonance IVIM-DWI imaging scans were performed after RFA in these patients.

#### 2.1.1 Inclusion and Exclusion Criteria

The inclusion criteria were as follows: the patient was diagnosed with HCC, received RFA therapy, and underwent MRI follow-up observation after surgery. The exclusion criteria were as follows: image artifacts were severe and seriously affected diagnosis and data measurement. For HCC tumors with a long diameter greater than 5 cm, RFA could not completely eliminate the tumor tissue, requiring a second surgery; such patients were not included in the study after the initial RFA.

#### 2.1.2 Diagnostic Criteria for Recurrent HCC

If one of the following conditions is met, recurrent HCC will be diagnosed. The lesion was confirmed as HCC by puncture or partial surgical resection. The lesion was diagnosed according to the European Association for the Study of the Liver (EASL) 2018 standard [1]. The patient had a history of liver cirrhosis. Nodules with a diameter of ≥ 2 cm could be diagnosed as HCC by performing enhanced CT or MRI, and ≥ 1 examination result showed “wash-in and wash-out” enhancement. Nodules with a diameter of 1 to 2 cm should undergo both enhanced CT and MRI examinations at the same time. If ≥ 1 examination result was positive, it could be diagnosed as HCC.

#### 2.1.3 **Diagnostic Criteria for** the **Inflammatory Reaction Zone**

If all the following conditions are met, we will diagnose the inflammatory reaction zone: (1) The enhanced scan of the lesion did not show signs of “wash-in and wash-out” similar to HCC. (2) During follow-up examination for 3–6 months, the lesion showed no enlargement or signs of space occupation. (3) During re-examination, the lesion’s arterial phase enhancement had decreased, matching the enhancement level of the surrounding liver tissue. (4) If it was an HCC patient with an increase in alpha-fetoprotein before RFA, during the follow-up process, there was no abnormal increase in alpha-fetoprotein, and its level was reduced.

### 2.2 Inspection Method

#### 2.2.1 Imaging Equipment

A Siemens 3.0T magnetic resonance imaging machine (Skyra MR 750) and an 18-channel abdominal phased array coil were used. The patient fasted and was not allowed to drink water for 6 to 8 hours before the examination. The patient was also given breath training before the examination.

#### 2.2.2 MRI Scanning Method

The scan was centered on the lesion, ranging from the upper margin to the lower margin of the liver. All patients underwent routine unenhanced MRI and enhanced sequences (including axial T1WI, T2WI fat-saturated, and DWI), as well as IVIM imaging sequence scans. Detailed imaging parameters are described in Table **1**. Scanning conditions: T1WI adopted the axial VIBE (volume interpolated breath-hold examination)-DIXON sequence. T2WI used axial respiratory-triggered T2 fat-saturated imaging. DWI used two b-values (b = 50, 800 s/mm^2^). The IVIM imaging sequence used multiple b-value SE-EPI sequences, with a total of 12 b-values selected (b = 0, 20, 40, 60, 80, 100, 160, 200, 400, 800, 1000, 1200 s/mm^2^). When the b-value ranged from 0 to 200 s/mm^2^, the number of acquisitions was 2. When the b-values were 400, 800, 1000, and 1200 s/mm^2^, the number of acquisitions was 6, 6, 10, and 12, respectively.

### 2.3 Quantitative Assessment

The images were diagnosed by three senior radiologists who had more than ten years of experience in liver MRI diagnosis. If the images were severely deformed, blurred, or had obvious respiratory motion artifacts, the examinations were not included in the study. The consistency of the measured values of the three observers was very good (intraclass correlation coefficient > 0.90) (Table **2**). By post-processing the data collected by the MR IVIM imaging sequence, three parameter maps could be obtained: the D parametric map, the D* parametric map, and the proportion of D* (f) parametric map.

The principle of selecting the ROI was as follows: recurrent HCC lesions and inflammatory reaction zones were clearly displayed on the D parameter map, but sometimes they were not clearly displayed on the D* and f parameter maps, which affected the accuracy of ROI placement. We used the multi-screen cross-reference function of the Siemens workstation to select the same scan slice for each IVIM parameter map. Then we determined the exact position of the lesion on the D parameter map and placed the ROI; in this way, an ROI of the same size would be automatically placed at the same position on the other parameter maps. This guaranteed an accurate ROI placement.

To avoid errors from biased ROI position selection, data for each lesion were measured three times and averaged. Then another ROI was placed in the normal hepatic parenchymal area around the lesion, with a size of about 100 mm^2^, measured three times at each position and the average was calculated. The ROI was positioned in the normal liver tissue surrounding the tumor, avoiding major blood vessels, bile ducts, and artifacts.

**Fig. (2A-F):**
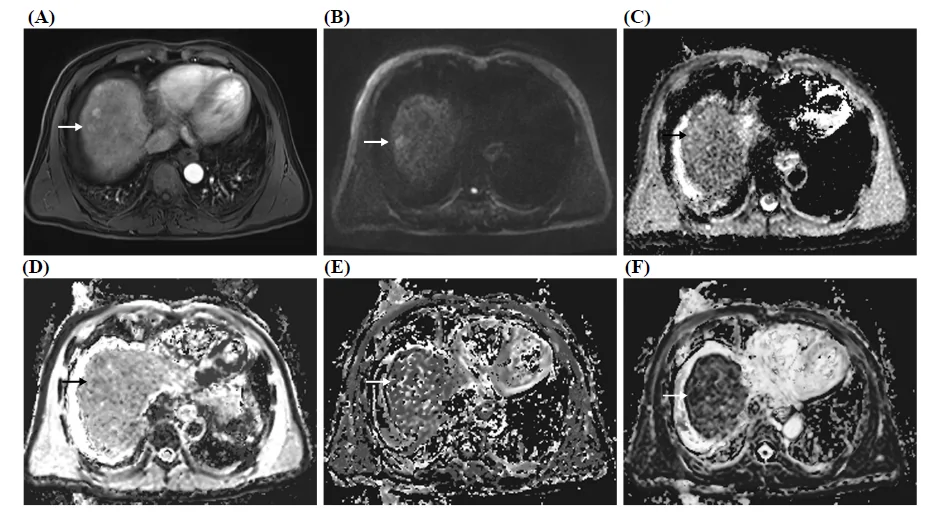
MR images in a 62-year-old man with recurrent HCC. Axial (**A**) enhanced fat-suppressed T1-weighted MR image shows a residual enhancing lesion with a nodular pattern (arrow) in the liver. Axial (**B**) DWI map shows a high homogeneous signal intensity. Axial (**C**) **ADC** map shows a low homogeneous signal intensity. Axial (**D**) * D* map shows a low homogeneous signal intensity. Axial (**E**)  D* map shows a heterogeneous isointense signal. The axial (**F**) f map shows a low, homogeneous signal intensity. The calculated mean values of **ADC**, D, D*, and *f* for the recurrent HCC were 0.68×10^-3^ mm^2^/s, 0.88×10^-3^ mm^2^/s, 46×10^-3^ mm^2^/s, and 22%, respectively.

**Fig. (3A-F):**
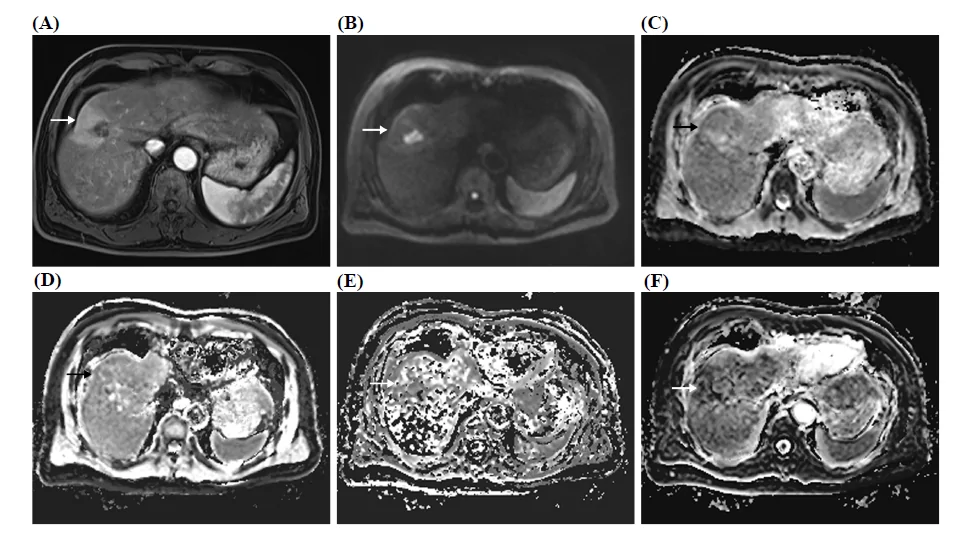
MR images in a 57-year-old man with an inflammatory reaction zone. Axial (**A**) enhanced fat-suppressed T1-weighted MR image shows the lesion with a peripheral rim of enhancement (arrow). Axial (**B**) DWI map shows a slightly higher heterogeneous signal intensity. Axial (**C**) **ADC** map shows a slightly lower heterogeneous signal intensity. Axial (**D**) * D* map shows a slightly lower heterogeneous signal intensity. Axial (**E**)  D* map shows a heterogeneous isointense signal. The axial (**F**) f map shows a heterogeneous, isointense signal. The calculated mean values of **ADC**, D, D*, and *f* for the recurrent HCC were 1.13×10^-3^ mm^2^/s, 0.85×10^-3^ mm^2^/s, 38×10^-3^ mm^2^/s, and 21%, respectively.

Among the 79 patients, 86 HCC lesions that met the inclusion criteria were identified and treated with RFA. During follow-up, 128 abnormally enhanced lesions were detected at the periphery of the RFA-treated area (Table **3**). Based on our diagnostic criteria, 61 lesions (47.7%) were diagnosed as recurrent HCC and 67 lesions (52.3%) were diagnosed as inflammatory reaction zones (Figs. **2** and **3**).

### 2.4 Statistical Analysis

We used the intraclass correlation coefficient to evaluate the repeatability of the measured values between different measurers. ICC < 0.40 means poor repeatability; 0.40 < ICC < 0.75 means good repeatability; ICC > 0.75 means very good repeatability. First, we used the one-sample Kolmogorov-Smirnov test to test the normality of the data (*P* > 0.10 indicates a normal distribution). The mean ± standard deviation (SD) was used to present continuous quantitative variables (*e.g*., D, D*, and f), whereas categorical variables (*e.g*., gender) were displayed as counts with percentages. Analysis of variance (one-way ANOVA) was used to compare differences in continuous variables such as D, D*, and f, and multiple comparisons between groups were performed using the Dunnett T3 test. The area under the ROC curve (AUROC) was used to measure how effectively the IVIM-DWI parameters could distinguish between recurrent HCC and the inflammatory reaction zone. The optimal cutoff values were identified from the highest Youden index. Statistical analysis was performed using SPSS (version 16.0, SPSS, Inc., USA). For all tests, *P* < 0.05 was considered statistically significant.

## 3 RESULTS

A total of 79 patients met the criteria of this study, including 69 males and 10 females. The patients were aged from 36 to 84 years, with an average of 59 ± 9.33 years. Among the 79 patients, there were 68 cases (86.1%) of hepatitis B, 6 cases (7.6%) of alcoholic cirrhosis, 4 cases (5.1%) of hepatitis C, and 1 case (1.3%) of drug-induced cirrhosis. Individuals with hepatitis had been living with the condition for several years. At the time HCC was detected, the average duration since the onset of hepatitis was 21.11 years. Among the 61 patients diagnosed with HCC, 48 (78.69%) exhibited elevated alpha-fetoprotein levels, with the normal range being 0–9 ng/ml. The average alpha-fetoprotein level at the time of HCC detection was 225.80 ng/ml. The minimum value was 16 ng/ml, and the maximum value was 1000 ng/ml. The consistency of the measured IVIM parameter values across various lesions and tissues among the three measurers was exceptional.

### 3.1 One-way Analysis of Variance of IVIM-DWI Parameters of Hepatic Parenchyma, RFA Lesion, Inflammatory Reaction Zone, and Recurrent HCC

All the data for the IVIM-DWI parameters followed a normal distribution.

Significant differences were observed in the D, D*, and f values within the hepatic parenchyma, RFA lesion, inflammatory reaction zone, and recurrent HCC (all *P*-values <0.001). Multiple comparative analysis results (Table **4**).

### 3.2 Comparison of the IVIM Parameters in the Recurrent HCC and the Inflammatory Reaction Zone by the ROC Curve

#### 3.2.1 Comparison of the *D* of the Recurrent HCC and the Inflammatory Reaction Zone

The AUROC was 0.95, *P* < 0.001. The findings indicated a statistically significant difference in D between recurrent HCC and the inflammatory reaction zone. Compared with the inflammatory reaction zone, the D of recurrent HCC was lower. With a cut-off value of 0.79 × 10^−3^ mm^2^/s, D had a sensitivity of 86.9%, a specificity of 94.0%, and a positive predictive value of 93.0%.

#### 3.2.2 Comparison of D* of the Recurrent HCC and the Inflammatory Reaction Zone

The AUROC curve was 0.80, *P*<0.01. The findings indicated a statistically significant difference in D* between recurrent HCC and the inflammatory reaction zone, with recurrent HCC having a lower D*. With a cut-off value of 34.75×10^-3^mm^2^/s, D* had a sensitivity of 62.3%, a specificity of 92.5%, and a positive predictive value of 88.4%.

#### 3.2.3 Comparison of the *f* of the Recurrent HCC and the Inflammatory Reaction Zone

**Fig. (4):**
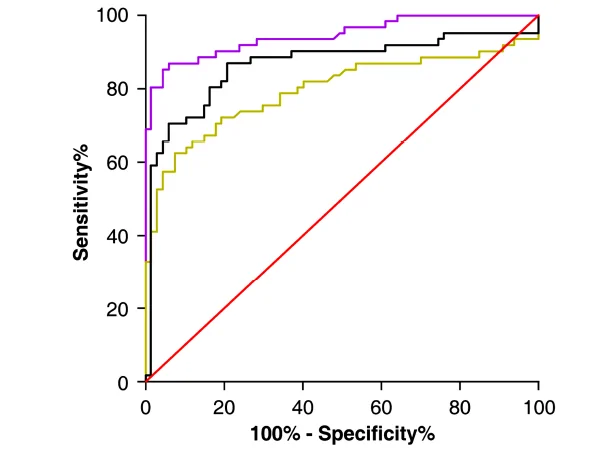
Lines (purple, yellow, and black) of the ROC curve were comparison of the IVIM-DWI parameters between the recurrent HCC and the inflammatory reaction zone (D, D*, and f, respectively), and the areas under the ROC curve were 0.95, 0.79, and 0.85, respectively.

The AUROC curve was 0.86, *P*<0.01. The findings indicated a statistically significant difference in f between recurrent HCC and the inflammatory reaction zone, with recurrent HCC having a lower f. With a cut-off value of 21.47%, *f* had a sensitivity of 86.9%, a specificity of 79.1%, and a positive predictive value of 86.9% (Table **5** and Fig. **4**)

## 4 DISCUSSION

The inflammatory reaction zone is an unusually intensified region surrounding the ablation area, resulting from heat-induced inflammatory swelling or granulation tissue growth in the adjacent tissue. Inflammatory reaction zones were observed in 89% of cases at 1 month, 56% between 1 and 3 months, and 22% at 6 months [15]. When a patient undergoes an enhanced magnetic resonance examination, both the inflammatory reaction zone and recurrent HCC will be enhanced. Therefore, the inflammatory reaction zone may cover recurrent lesions, which may affect the assessment of the effect of RFA treatment.

As a functional imaging technique, DWI, which mainly depends on the movement of water molecules in tissue, is widely recognized for its role in liver disease diagnosis [16]. There are two main factors that affect the movement of water molecules in living biological tissues. One is the diffusion motion of water molecules, namely Brownian motion, which is related to the physical properties of tissues and can reflect the physiological and pathological state of the local microscopic tissue structure. The second is the microcirculation of blood flow in the capillary network, that is, perfusion. Therefore, the ADC (apparent diffusion coefficient) of living tissue obtained by DWI imaging is affected by many factors, the most important of which is the attenuation of the phase of the blood perfusion component, which makes the measured ADC higher than the true ADC [17], especially in hyperperfused tissues (such as the liver). Le Bihan and other scholars first proposed the DWI imaging method based on IVIM [18], which can separate tissue diffusion motion components and blood flow perfusion components through a double-exponential model calculation [11].

The map biomarkers obtained by IVIM imaging include the true diffusion coefficient D (slow ADC), pseudo-diffusion coefficient D* (fast ADC), and perfusion fraction f. D truly reflects the diffusion state of water molecules in tissue. D* is related to the microcirculation state of the capillary network and reflects the capillary flow velocity of tissue. f refers to the ratio of capillary volume to total tissue volume in voxels, and its value ranges between 0 and 1 [19]. Therefore, non-enhanced IVIM-DWI can simultaneously quantify changes in water molecule diffusion and microcirculation perfusion, thereby quantitatively evaluating the functional status of tissues after RFA of HCC. In this study, IVIM-DWI imaging was processed to generate D, D*, and f.

### 4.1 The Ability of *D* to Distinguish between Recurrent HCC and the Inflammatory Reaction Zone

Our research indicated that the D of recurrent HCC was lower than that of the inflammatory reaction zone. The optimal cutoff value can differentiate the two types of lesions.

Studies have shown that the D of solid lesions (HCC, hepatic hemangioma) is lower than that of cystic lesions, and the D of malignant tumors (HCC, liver metastases) is lower than that of benign tumors (hepatic hemangioma) [12], which is consistent with the results of this study. Because the density of cells in a solid tumor is significantly higher than that of a cystic lesion and the tissue gap is correspondingly reduced, the diffusion of water molecules in solid tumor tissue is more restricted than in cystic lesions. The D of malignant tumors is lower than that of benign tumors. This is considered to be related to the active growth of HCC, the increase in cell density, and the corresponding decrease in interstitial space. The inflammatory reaction zone, which is caused by the increase in vascular permeability after liver tissue is locally heated and by the temporary increase of water molecules in the interstitial spaces around blood vessels, has the same cellular components as hepatic parenchyma. From the research conclusions, this change has a significant impact on the diffusion of water molecules in the inflammatory reaction zone. Therefore, the difference between the D of the inflammatory reaction zone and hepatic parenchyma is also statistically significant. An animal experiment [14] demonstrated that the D and f values were significantly lower in viable rabbit liver VX2 tumors than in inflammatory reaction zones, which is consistent with the results of our study.

### 4.2 The Ability of D* to Distinguish between Recurrent HCC and the Inflammatory Reaction Zone

D* is significantly positively related to microvessel density and can reflect the density of microvessels in the lesion. For patients with contraindications to contrast agents, the microcirculatory functional status of the lesion can be analyzed by IVIM without using contrast agents [20].

Our research indicated a statistically significant difference in D* between recurrent HCC and the inflammatory reaction zone. The ROC curve shows that the optimal cutoff value has high diagnostic efficiency. After RFA of HCC, the internal tissues are necrotic and the blood vessel structure is destroyed, so in theory there is no microvascular perfusion. We measured the enhanced scan images and found that completely inactivated HCC tissue did not show abnormal enhancement. Therefore, the D* of RFA lesions is the lowest. The inflammatory reaction zone is a manifestation of inflammatory edema of liver tissue after heating. There is no variation in vascular composition between hepatic parenchyma and the inflammatory reaction zone. It is considered that this is the reason why the difference between the inflammatory reaction zone and hepatic parenchyma D* mean values is not statistically significant. HCC grows rapidly and is mainly supplied by the hepatic artery and lacks the normal structure of the portal vein. We believe that this is related to the decrease in HCC perfusion coefficient and microvessel density, leading to a decrease in its D* to lower than that of the inflammatory reaction zone.

### 4.3 The Ability of *f* to Distinguish between Recurrent HCC and the Inflammatory Reaction Zone

The f reflects the percentage of capillary volume in the voxel to the total tissue volume (0 < f < 1). Similar to D*, the blood flow velocity and blood volume of the lesion determine the value of f [19].

In this study, the f of the hepatic parenchyma and the inflammatory reaction zone were higher than that of other tissues, and the difference was statistically significant (*P* < 0.001). This is because relatively normal hepatic parenchyma contains a large number of blood vessels and fast, freely moving blood molecules, and the blood volume and blood flow velocity are higher than in other tissues. Because the blood vessels in the radiofrequency ablation lesion have been completely destroyed, it is basically necrotic tissue, which leads to its low f. Because HCC grows rapidly and is mainly supplied by the hepatic artery, it lacks a normal portal vein structure. It is considered that this is related to the decrease in its perfusion coefficient and microvessel density, which leads to a decrease in its f to lower than that of the inflammatory reaction zone.

## 5 LIMITATIONS OF THIS CASE STUDY

Firstly, in clinical practice, it is difficult to use liver biopsy every time to distinguish the inflammatory reaction zone from the recurrent tumor (multiple tumors, high risk of puncture complications, *etc*.). For lesions for which pathological diagnosis cannot be established, we adhere strictly to internationally recognized diagnostic standards or develop rigorous clinical criteria for diagnosis, and confirm the diagnosis through follow-up examinations. Secondly, during MRI scans performed with voluntary breathing, movement caused by respiration and cardiac contractions can lead to inaccuracies in measuring parameters related to liver lesion progression, especially in small lesions located in the left lobe of the liver. As a result, some lesions in the left lateral lobe of the liver were not included in the study, which may have an impact on the research findings.

## CONCLUSION

In conclusion, our study shows that IVIM-DWI has potential for early evaluation of RFA treatment efficacy in HCC. IVIM parameters may serve as valuable markers to distinguish viable tumors from the inflammatory reaction zone in post-RFA HCCs. Furthermore, in terms of diagnostic performance, D outperformed D*, and f. Our findings indicate that IVIM-DWI can distinguish recurrent HCC from the inflammatory reaction zone within 3 months, which is helpful for early radiofrequency ablation treatment of the tumor. In addition, IVIM parameters may be valuable markers for differentiating viable tumors from the inflammatory reaction zone in post-RFA HCCs. In the literature search, the authors did not find any relevant studies that used IVIM-DWI maps to evaluate the inflammatory reaction zone of RFA lesions.

## Figures and Tables

**Table 1:** 3.0-T MR imaging parameters.

Sequence	TR/TE (msec)	FA (degree)	Matrix	FOV	ST (mm)	IG (%)	AT
T1-VIBE-BH	4.11/1.28	12	195×320	325×400	3	0	15sec
T2-BLADE-RT	3000/79	150	320×320	380×380	6	20	190sec
DWI-SS-EPI	4800/68	180	128×128	400×400	5	30	110sec
IVIM-SS-EPI	2200/58	180	256×128	360×360	5	20	58sec

**Abbreviations**: TE=echo time, TR=repetition time, FA=flip angle, FOV=field of view, ST=section thickness, IG=intersection gap, AT=acquisition time, VIBE=volumetric interpolated breath-hold examination, BH=breath-hold, RT=respiratory triggered, DW=diffusion-weighted, SS-EPI=single-shot echo- planar imaging, IVIM=intravoxel incoherent motion.

**Table 2:** Consistency analysis results of IVIM parameters measured by three measurers.

Group	ICC of *D*	ICC of D*	ICC of *f*
RFAL	0.998	0.953	0.972
HP	0.993	0.996	0.998
RHCC	0.932	0.905	0.913
IRZ	0.925	0.916	0.904

**Abbreviations**: ICC, intraclass correlation coefficient; RFAL, Radiofrequency ablation lesion; HP, hepatic parenchyma; RHCC, recurrent HCC; IRZ, inflammatory reaction zone.

**Table 3:** Basic information of the patient's lesion.

Group	Recurrent HCC	IRZ	RFAL
Size (cm)	2.32 (0.66)	2.99(0.90)	4.70(1.45)
Location of Lesion n (%)			
Segment 8	9(14.8%)	11(16.4%)	14(16.5%)
Segment 5	15(24.6%)	8(11.9%)	16(18.8%)
Segment 7	19(31.1%)	23(34.3%)	22(25.9%)
Segment 6	11(18.0%)	16(23.9%)	19(22.4%)
Segment 4	4(6.6%)	7(10.4%)	10(11.8%)
Segment 3	2(3.3%)	1(1.5%)	3(3.5%)
Segment 1	1(1.6%)	1(1.5%)	1(1.2%)

**Note:** Data were expressed as mean (standard deviation) or quantity (%). **Abbreviations**: IRZ, inflammatory reaction zone; RFAL, Radiofrequency ablation lesion.

**Table 4:** IVIM-DWI parameters in different tissues.

Group	n	*D*(×10^-3^ mm^2^/s)	D*(×10^-3^ mm^2^/s)	*f* (%)
RFAL	86	1.33±0.51	25.36±12.02	12.75±6.97
HP	79	0.96±0.17	45.08±19.12	23.94±8.60
RHCC	61	0.65±0.16	31.34±11.54	18.16±5.82
IRZ	67	0.87±0.05	41.37±5.11	24.21±3.69

**Abbreviations**: IVIM-DWI=intravoxel incoherent motion diffusion-weighted imaging, HP=hepatic parenchyma, RFAL=radiofrequency ablation lesion, IRZ=inflammatory reaction zone, RHCC=recurrent HCC. **Note:** Data were expressed as mean ± standard deviation Post hoc comparison results: I- *D*: RFAL>HP>IRZ>RHCC, All *P*-Values <0.001. II- D*: [IRZ=HP]>RHCC>RFAL, *P*-Values: >0.05 and≤ 0.01. III- *f*: [IRZ=HP]>RHCC>RFAL, *P*-Values: >0.05 and <0.001.

**Table 5:** Results of ROC curve comparison of IVIM parameters of RHCC and IRZ: Diagnostic indices of selected cutoff points of ROC analyses.

Group	CV	Se (%)[95% CI]	Sp (%)[95% CI]	PPV (%)[95% CI]	NPV(%)[95%CI]
*D* (×10^-3^ mm^2^/s)	0.79	86.9[75.2-93.8]	94.0[84.7-98.1]	93.0[82.2-97.7]	88.7[78.5-94.7]
D* (×10^-3^ mm^2^/s)	34.74	62.3[50.0-74.1]	92.5[82.7-97.2]	88.4[74.1-95.6]	72.9[62.0-81.7]
*f* (%)	21.47	86.9[75.2-93.8]	79.1[67.1-87.7]	79.1[67.1-87.7]	86.9[75.2-93.8]

**Abbreviations**: IVIM = intravoxel incoherent motion, RHCC = recurrent HCC, IRZ = inflammatory reaction zone, ROC = receiver operating characteristic, CV = cutoff value, Se = sensitivity, Sp = specificity, 95% CI = 95% confidence interval, PPV = positive predictive value, NPV = negative predictive value.

## Data Availability

The data that support the findings of this study are available from the corresponding authors [Z.P] and [E.J] upon reasonable request.

## LIST OF ABBREVIATIONS

IVIM-DWI: Intravoxel Incoherent Motion Diffusion-weighted Imaging
MRI: Magnetic Resonance Imaging
HCC: Hepatocellular Carcinoma
RFA: Radiofrequency Ablation
ROC: Receiver Operating Characteristic
CT: Computed Tomography
AUROC: Area Under the ROC Curve
TE: Echo Time
TR: Repetition Time
FA: Flip Angle
FOV: Field of View
ST: Section Thickness
IG: Intersection Gap
AT: Acquisition Time
VIBE: Volumetric Interpolated Breath-hold Examination
BH: Breath-Hold
RT: Respiratory Triggered
SS-EPI: Single-shot Echo- planar Imaging
ICC: Intraclass Correlation Coefficient
RFAL: Radiofrequency Ablation Lesion
HP: Hepatic Parenchyma
RHCC: Recurrent HCC
IRZ: Inflammatory Reaction Zone
CV: Cutoff Value
Se: Sensitivity
Sp: Specificity
CI: Confidence Interval
PPV: Positive Predictive Value
NPV: Negative Predictive Value

## STANDARDS OF REPORTING

STROBE guidelines were followed.
